# Immunoproteomic analysis of *Plasmodium falciparum* antigens using sera from patients with clinical history of imported malaria

**DOI:** 10.1186/1475-2875-12-100

**Published:** 2013-03-18

**Authors:** Rita M Costa, Fátima Nogueira, Karina P de Sousa, Rui Vitorino, Marcelo S Silva

**Affiliations:** 1 Centre for Malaria and Tropical Diseases, Instituto de Higiene e Medicina Tropical, Universidade Nova de Lisboa, Rua da Junqueira, 100, Lisbon, 1349-008, Portugal; 2 QOPNA, Department of Chemistry, University of Aveiro, Aveiro, Portugal

**Keywords:** Plasmodium falciparum, Imported malaria, Antibody response, Malaria antigens

## Abstract

**Background:**

The malaria caused by *Plasmodium falciparum* remains a serious public health problem in the world, due largely to the absence of an effective vaccine. There is a lack of information on the structural properties and antigens capable of activating the immunological mechanisms for the induction of protective immunity. Therefore, the objective of this study is to evaluate the serological reactivity of sera from individuals with imported malaria and identify major immunogenic proteins.

**Methods:**

The study was conducted in 227 individuals with imported malaria and 23 healthy individuals who had never been in areas endemic for malaria. The determination of anti-*P. falciparum* IgG antibodies was performed by an ELISA validated and optimized for this study. Sera showing higher reactivity to anti-*P. falciparum* by ELISA were analysed by immunoblotting and immunogenic proteins were identified by mass spectroscopy.

**Results:**

The results of anti-*P. falciparum* antibodies research by ELISA indicates 78 positive, 137 negative and 12 indeterminate sera. Analysis of immunoblotting demonstrated a consistent pattern with respect to immunoreactivity of antigens with molecular weights in the range of 40 to 60 kDa. Between 40 and 60 kDa six immunogenic proteins were identified: elongation factor-1 alpha (EF-1α), protein disulphide isomerase (PDI); phosphoglycerate kinase (PGK); 78 kDa glucose-regulated protein homologue (GRP-78); rhoptry-associated protein 2 (RAP-2) and rhoptry-associated protein 3 (RAP-3).

**Conclusions:**

It was identified immunogenic proteins essential for parasite survival in the host, two of which (RAP-2 and RAP-3) are already described in the literature as proteins that play an important role in the invasion of erythrocytes by extracellular merozoites.

## Background

Malaria is an infectious disease caused by unicellular, obligate, intracellular, protozoan parasites belonging to the genus *Plasmodium* and the main form of transmission is through the bite of the female Anopheles mosquito. There are over 200 species in the genus *Plasmodium*, but only five species of malaria parasites infect humans: *Plasmodium falciparum*, *Plasmodium ovale*, *Plasmodium malariae, Plasmodium vivax* and *Plasmodium knowlesi*. *Plasmodium falciparum* is the most virulent of the human malaria parasites: it can cause severe malaria, a complication that is often fatal and is responsible for the bulk of the malaria-related mortality, particularly in African infants [1].

Malaria is currently endemic in the tropical zones with extensions into the subtropical regions of Asia, Africa, South and Central America. According to the 2011 World Malaria Report, there were an estimated 216 million episodes of malaria and 655,000 worldwide malaria deaths in 2010, 91% of which were in Africa [2]. Approximately 86% of malaria deaths globally were children less than five years of age from sub-Saharan Africa [2].

Malaria remains one of the most serious public health problems not only in endemic countries but also in non-endemic areas where the increasing number of imported malaria cases is worrying [3]. In developed countries, imported malaria predominates in tourists and immigrants. Every year, approximately 125 million international travellers visit malaria-endemic areas and 30,000 of them contract the disease [4,5]. Portugal maintains a close relationship with its former colonies for several reasons and so malaria must be suspected in travellers that return from those countries. Malaria is a notifiable disease in Portugal and about 50 cases are reported annually to the National Public Health System [4,6]. Non-immune people (from non-endemic countries) who stay for some time in malaria-affected areas are at increased risk of having severe forms of the disease and can develop symptoms of *P. falciparum* malaria within a month after leaving the endemic area [7]. In non-endemic countries for malaria, the diagnosis and adequate treatment is delayed due to the fact that malaria is infrequent in these countries, which can make these cases fatal to patients.

The life cycle of malaria parasites is complex. The invasion of erythrocytes by *Plasmodium* merozoites is a multistep process of specific protein interactions between the parasite and the red blood cell. The first step is the initial recognition and reversible merozoite attachment to the erythrocyte followed by its apical reorientation, irreversible junction formation between the apical end of the merozoites and finally entry into the red cell in a parasitophorous vacuole. This stage of the life cycle is regarded as an attractive target for the development of interventions to combat malaria since the invasion of erythrocytes by the merozoites requires specialized protein expression, such as merozoite surface protein 1 (MSP-1) and a series of highly specific molecular interactions.

The emergence of resistant strains of the parasite, the spread of mosquito strains resistant to insecticides and lack of effective vaccines against malaria are the main factors hampering the fight against the disease. Given the limited exposure of these proteins to the immune system of the host, the antibodies are likely to be the main form of immunity against merozoites [8]. Epidemiological and experimental evidence has revealed the crucial importance of antibody-mediated effector mechanisms directed predominantly at antigens of blood stage parasites [9]. In a similar study, gamma globulin of adults in West Africa was administered to individuals of East Africa with serious infections by *P. falciparum*, which led to the abrupt reduction of parasite density and recovery of clinical disease [10]. In Thai patients, the protective effect of IgG antibodies against African *P. falciparum* was also demonstrated by passive transfer [11], and antibodies to immune African individuals are capable of controlling the erythrocytic stage of the parasite in Saimiri monkeys [12]. To date, the exact mechanism of protection, whereby the malaria-specific antibodies interfere with the development and/or proliferation of asexual stages of *P. falciparum*, is still not accurately known, and the antigens targeted by protective antibodies remain a topic for discussion.

Immunization with antigens of the erythrocytic phase, particularly antigens from merozoites, has been shown to be protective in various animal models and to have some protective effect in humans [13]. Studies in human and in animal models have shown that the immune response directed to erythrocytic stage antigens may be protective and facilitate the control of infection [14]. It is known that in endemic areas naturally IgG antibodies mediate acquired immunity against *P. falciparum*.

Despite the global importance of *P. falciparum*, most of their proteins have not been yet characterized experimentally. Therefore, the main objective of this study is to evaluate the serological reactivity of serum samples from individuals, mainly from African countries, who have potentially been exposed to *P. falciparum*, compared to native parasite antigens and subsequent identification of major immunogenic proteins.

## Methods

### Study population

Sera recruited for this study of patients with imported malaria, as well as negative sera (healthy Portuguese individuals who have never been in malaria-endemic countries) were obtained at the Clinical Unit for Tropical Diseases (IHMT, Portugal).

The population for this study consisted of 227 individuals with imported malaria and 23 healthy individuals (residents in Portugal). Most are adults who were in different endemic regions of Africa, including Angola, Guinea, Mozambique, Sao Tome, Cape Verde, Madagascar, Gabon, Congo, Tanzania and Morocco. Some sera were from individuals who had been in Brazil, Ecuador, India, Indonesia, Thailand and Haiti. As a negative control, a group of 23 sera from healthy Portuguese individuals, who had never been in endemic countries, was used.

### Consent

The institutional Ethics Committee at the Instituto de Higiene e Medicina Tropical, Lisbon, Portugal, approved this study protocol*.* Written informed consent was obtained from the patient for serological assays for malaria and publication for this manuscript (Additional files 1 and 2).

### Cultured parasites

3D7 *P. falciparum* clones were cultured at 5% haematocrit, 37°C and atmosphere with 5% of CO_2_ as described by [15,16]. Human serum was replaced with 0.5% AlbuMAXII (Invitrogen ™) in the culture medium. When parasitaemia reached about 8-10%, cultures were harvested for parasite protein extraction.

For the extraction of total proteins of *P. falciparum* cultures were centrifuged at 5,000 rpm for five minutes at room temperature, and the supernatant was discarded. Pellet was washed with PBS once, resuspended in equal volume of PBS and a solution of saponin 0.5% in PBS and incubated for 10 minutes at 4°C. Subsequently the mixture was centrifuged at 10,000 rpm at 4°C for 10 minutes, the supernatant discarded and parasites washed three times with ice-cold PBS. For the extraction of all proteins of the parasite lysis buffer was added (0.5% NP-40, 120 mM NaCl, 50 mM Tris–HCl pH 7.8), homogenized thoroughly in the vortex and centrifuged for 15 minutes at 10,000 rpm at 5°C. The supernatant was recovered, discarding the haemozoin. Proteins were quantified by the Bradford method.

### Determination of anti-*Plasmodium falciparum* antibodies

It was used the total protein of *P. falciparum* antigen for the search of IgG antibodies in serum from patients with imported malaria. Briefly, 96-well microplates (BD FALCON^TM^ - EUA) were coated with 100 ng/well of the antigen diluted in bicarbonate buffer (0.1 M, pH 8.5), overnight at 4°C. All ELISAs were carried out using a 100 μL reaction mixture volume. After incubation, the plates were washed three times with 200 μL of wash buffer (PBS containing 0.05% Tween 20) per well and the wells were blocked with 5% low-fat milk in PBS (pH 7.2) for one hour at room temperature with orbital stirring. Plates were washed three times with 200 μL/well with wash buffer. Then the plates were incubated with 100 μL/well of diluted sera, for one hour at room temperature with orbital stirring. After incubation with serum samples, the plate was washed five times with 200 μL of wash buffer. For detection of human anti-*P. falciparum* antibodies, the plate was incubated with 100 μL/well of anti-human horseradish peroxidase (HRP)-conjugated IgG secondary antibody (Sigma-USA) for one hour at room temperature with orbital agitation. Then, five washings were made, each with 200 μL/well of wash buffer. To reveal the presence of conjugate the plate was incubated with 100 μL/well of substrate solution (10 mL citrate buffer pH 5.0 with 10 mg OPD, and 10 μL of hydrogen peroxide 30% v/v), for 30 minutes at room temperature and protected from light. To stop the reaction, it was used 4.0 N sulphuric acid. The cut-off value was defined as a mean of OD from negative control, plus twice standard deviation (Cut-off = m + 2SD).

### Protein characterization and immunoblotting

*Plasmodium falciparum* proteins were separated by SDS-PAGE with a 12% polyacrylamide gel. The determination of the protein’s molecular mass was estimated by comparison with a commercial HyperPAGE® (Bioline, UK) molecular weight marker. After SDS-PAGE, the gel was withdrawn from the cassette and the gel was equilibrated in transfer buffer for 20 minutes to remove the excess SDS. The membrane and the filter paper used for transfer were equilibrated in transfer buffer for 10 minutes. The antigenic proteins were transferred to a PVDF membrane, previously activated in methanol for five seconds.

The electroblotting was performed in a semi-dry system (BIO-RAD, USA) at 15 V for 15 minutes. After transfer of proteins from the gel to the membrane, the membrane was washed twice with 15 mL of PBS for 10 minutes each. This was followed by blocking of the membrane with 15 mL blocking solution (3% BSA w/v in PBS buffer) for one hour at room temperature. To remove the excess BSA, the membrane is washed three times, twice with 20 mL PBS-0.5% Tween for 10 minutes each, and once with 15 mL of PBS for 10 minutes. For immunodetection of antigens on the membrane were used serum samples from patients with higher reactivity observed by ELISA (n = 32). As negative controls, it was used sera from patients who had never been in contact with the parasite or in malaria-endemic areas. This was followed by incubating the membrane with 10 mL of primary antibody (1:1,000 in blocking solution) for one hour at room temperature with orbital agitation. After incubation with primary antibodies the membrane was washed three times, twice with 20 ml of PBS-0.5% Tween for 10 minutes each, and once with 15 mL of PBS for 10 minutes. For detection of the antibody-antigen complex the membrane was incubated with 8 mL of secondary antibody anti-IgG-HRP (Sigma-USA) diluted 1:6,000 in blocking solution for one hour at room temperature with orbital agitation. After the incubation period, the membrane was washed five times with 20 mL PBS-0.5% Tween for 10 minutes each. To reveal the presence of the conjugate the membrane is incubated with 15 mL of developing solution (7.5 mg diaminobenzidine in PBS with 0.02% H_2_O_2_) at room temperature until colour development. The reaction was quenched with 20 mL of distilled water. After stopping the reaction membrane was dried and stored for later use in the identification of immunogenic proteins by mass spectroscopy.

### Protein identification by mass spectrometry

Protein bands were excised manually from the SDS-PAGE gel distained with 25 mM ammonium bicarbonate/50% acetonitrile and dried under vacuum (SpeedVac®, Thermo Savant, USA).

The dried gel pieces were rehydrated with 25μL of 10 μg/mL trypsin (Promega, USA) in 50 mM ammonium bicarbonate and digested overnight at 37°C. Tryptic peptides were extracted from the gel with 10% formic acid/50% acetonitrile and dried in a vacuum concentrator, resuspended in 10μL of a 50% acetonitrile/0.1% formic acid solution and mixed (1:1) with a matrix consisting of *a*-cyano-4-hydroxycinnamic acid. Aliquots of samples were spotted onto the MALDI sample target plate. Mass analysis of peptides of each sample was performed with a 4800 MALDI-TOF/TOF Analyzer (ABSciex) in automatic mode with the following settings: for the MS data, m/z range 800 to 4,500 with an accelerating voltage of 20 kV and delayed extraction, peak density of maximum 50 peaks per 200 Da, minimal S/N ratio of 10 and maximum peak at 65. Spectra were internally calibrated with peptides from trypsin autolysis (M + H + = 842.509, M + H + = 2211.104).

For the MS/MS data, fragment selection criteria were a minimum S/N ratio of 6, a maximum number of peaks set at 65 and peak density of maximum 50 peaks per 200 Da. For each precursor selected for MS/MS analysis, fragment mass values in the range from 60 Da to 10 Da below precursor mass were used to peptide identification. Protein identification was assigned by peptide mass fingerprinting and confirmed by MS/MS analysis. Mascot (v2.1, Matrixscience) was used for protein identification running on GPS software (Applied Biosystems). Searches were performed against the NCBi (October2011) under all taxonomic categories and the following parameters: (i) two missed cleavages by trypsin; (ii) mass tolerance of precursor ions 25 ppm and product ions 0.3 Da; (iii) carboxymethylated cysteines; and, (iv) oxidation of methionine as variable modification. Protein identifications were considered as reliable when the individual ion score for each peptide had a minimum individual score of 95% and a minimum sequence tag of four amino acids (five consecutive peaks in the MS/MS spectrum).

## Results

In this study, an anti-*P. falciparum* immunoassay (ELISA) was validated and optimized to distinguish the more reactive sera with regard to the presence of specific anti-*P. falciparum* IgG antibodies in human serum samples. All sera of patients with imported malaria were analysed for antibodies of the IgG type, at a dilution of 1:40,000. These patients were actively recruited in the Clinical Unit for Tropical Diseases (IHMT, Portugal).

To differentiate positive from negative sera a limit point (cut-off) was defined. To determine the cut-off value it was used the 23 sera from healthy Portuguese individuals (control group) who had never been in malaria-endemic areas. The cut-off value was defined as a mean of OD from negative control, plus two standard deviation (cut-off = m + 2SD); at a dilution of 1:40,000. The weight that the standard deviation (SD) may have on the cut-off value depends on the sensitivity and specificity of this cut-off value will give the method [17]. This was chosen weighting (2SD) because with this cut-off value all negative sera have an absorbance value less than or equal to the cut-off value chosen for dilution.

**Figure 1:**
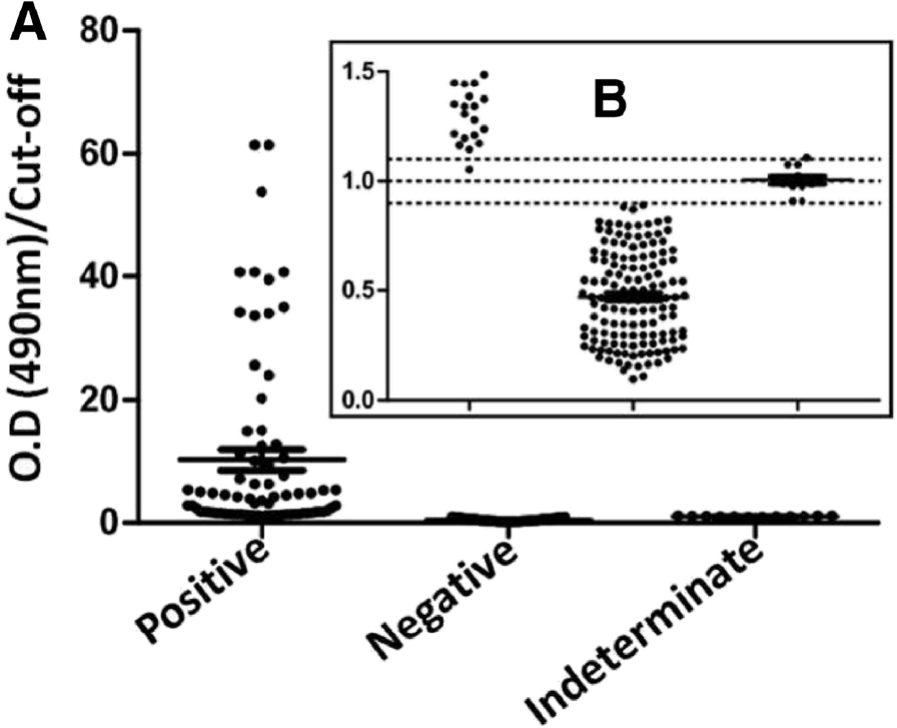
**Results of the study of reactivity of sera from patients with acute imported malaria by anti-*Plasmodium falciparum *ELISA. A** - IgG specific antibodies were searched within a total of 227 sera. Positive n = 78; negative n = 137 and indeterminate n = 12. **B** – Zoom in on O.D/Cut-off ratio = 1.

For the analysis of the results, it was used as discernible criteria the ratio between the absorbance/cut-off; all sera with this ratio above 1.1 were considered positive for anti-*P. falciparum* antibodies (Figure 1). Apart from these groups, another group was set up, the indeterminate group, which were all sera with a value of absorbance within a range of cut-off ± 10% of cut-off value. The results of anti-*P. falciparum* antibodies research by ELISA indicates 78 positive, 137 negative and 12 indeterminate sera.

Through fractionation of antigens of *P. falciparum* identified by SDS-PAGE associated with the immunoblotting technique, it was identified specific components of parasite that may be associated with acute infection, since the tested sera were from patients with imported acute malaria.

**Figure 2:**
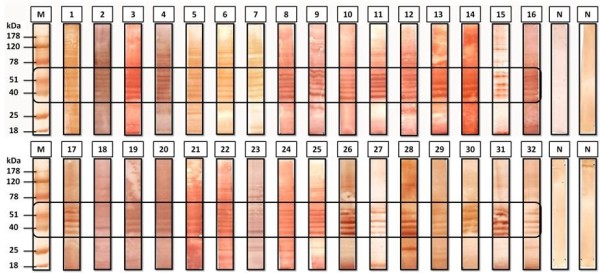
**Immunoblotting profiles of the reactive sera by anti-*Plasmodium falciparum *ELISA.** Each strip contains approximately 14 μg of *P. falciparum*. M - Molecular mass marker Hyper PAGE®, N - negative control sera.

**Figure 3:**
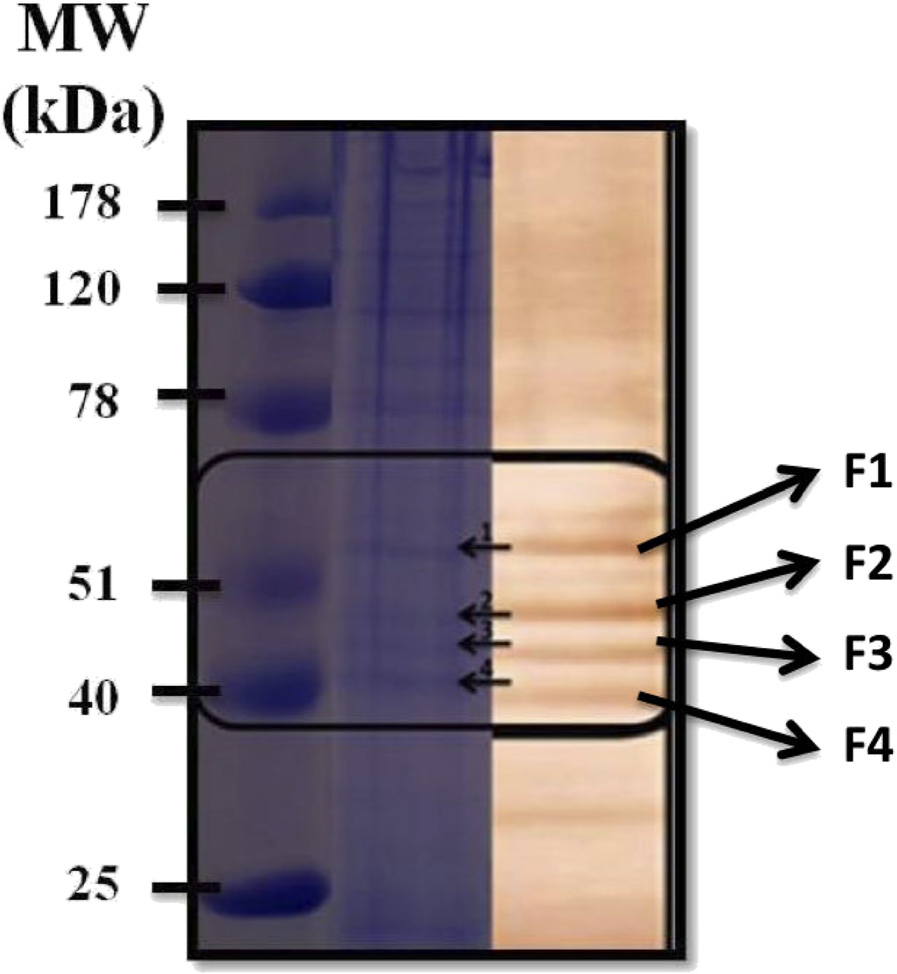
**Result of protein identification by mass spectrometry.** Six proteins were identified from a previous SDS-PAGE. F1 - elongation factor-1 alpha (EF-1α), protein disulphide isomerase (PDI); phosphoglycerate kinase (PGK); 78 kDa glucose-regulated protein homologue (GRP-78); rhoptry-associated protein 2 (RAP 2) and rhoptry-associated protein 3 (RAP 3).

Analysis of immunoblotting profiles of the 32 most reactive sera by ELISA demonstrated a consistent pattern with respect to immunoreactivity of antigens with molecular weights in the range of 40 to 60 kDa (Figure 2). Between 40 and 60 kDa, was identified four main bands in the membranes that were analysed by mass spectrometry (Figure 3 and Additional file 3) and it was identified six immunogenic proteins: elongation factor-1 alpha (EF-1α), protein disulphide isomerase (PDI); phosphoglycerate kinase (PGK); 78 kDa glucose-regulated protein homologue (GRP-78); rhoptry-associated protein 2 (RAP 2) and rhoptry-associated protein 3 (RAP 3) (Additional file 3).

## Discussion

In order to achieve the proposed objectives, it was initially cultivated the parasite *P. falciparum* (3D7) in an *in vitro* system of red blood cells (RBCs), followed by the extraction of total native proteins from the parasite and its quantification by the Bradford method. It was also used enzyme immunoassays (ELISA) to distinguish a serologically negative from a positive population, compared to a control group of healthy individuals (n = 23) from Portugal, who had never been in areas that are endemic to malaria. Sera displaying greater reactivity to anti-*P. falciparum* antibodies by ELISA were analysed by immunoblotting. The analysis of the blots profiles demonstrated a consistent pattern with respect to the immunoreactivity of antigens with molecular weights ranging from 40 to 60 kDa (Figure 2).

The life cycle of *P. falciparum* is complex since its nature requires expression of multiple specialized proteins necessary for the invasion of different types of cells as well as to survive in both invertebrate (mosquito) and vertebrate (human) host organisms [18]. Due to the complexity of the life cycle, each life stage expresses a number of antigenic proteins that can induce an immune response and which presents a real challenge for vaccine design [19].

It was observed that the immunoreactivity of the sera from patients potentially exposed to *P. falciparum* corresponds to some of the antigens prevalent in total protein extracts from parasites and six immunogenic proteins were identified by mass spectrometry (Figure 3 and Additional file 3): elongation factor-1 alpha (EF-1α); protein disulphide isomerase (PDI); phosphoglycerate kinase (PGK); 78 kDa glucose-regulated protein homologue (GRP-78), rhoptry-associated protein 2 (RAP-2) and rhoptry-associated protein 3 (RAP-3).

Eukaryotic elongation factor 1A (eEF1A) plays a central role in protein synthesis, cell growth and morphology [20,21]. Malaria parasite possesses two identical genes encoding eEF1A (*eef1aa* and eef1ab). Studies using *pbeef1a P. berghei* mutants that lack an eEF1a gene show that the level of eEF1A production affects the proliferation of blood stages and parasite fitness [21]. Further attenuation of parasites through elongation of the cell cycle by affecting protein synthesis machinery, might be used for testing the feasibility of the creation of attenuated live parasites as vaccine [21].

Proteins disulfide isomerases (PDI) are a family of redox chaperones that are implicated in protein unfolding and trafficking across the endoplasmic reticulum (ER) and towards the cytosol, a thiol-based redox locus for antigen processing [22].

The major pathway for glucose metabolism in *P. falciparum*-infected RBCs is anaerobic glycolysis. PGK is one of the key enzymes of this pathway as it generates ATP [23]. The GRP-78 is a heat shock-related protein and may play a role in the adaptation of parasites during their complex life cycle [24]. The stages of the parasite that grow and multiply in RBCs cause all the pathological effects and accordingly the invasion of RBCs is one of the most important steps in the parasite life cycle.

The rhoptries are specialized secretory organelles located at the apical end of merozoites, the form of the parasite that invades RBCs. Its contents include proteins that have been implicated as having a role in the invasion process such as RAP-2 and RAP-3 [25,26]. RAP-1 controls RAP-2 or RAP-3 transport toward the rhoptries during invasion, when the gene encoding RAP-1 is interrupted, the traffic of either of the other two proteins is affected and such proteins remain trapped in the endoplasmatic reticule. Invasion does not become inhibited when the gene encoding RAP-3 is interrupted but does become reduced to some degree. These observations suggest that the loss of RAP-3 is compensated for by the presence of RAP-2 and *vice-versa*. Several studies have demonstrated the importance of responses mediated by antibodies to rhoptry proteins in protection against malaria. *In vitro* studies showed that monoclonal antibodies directed against RAP-2 provide substantial inhibition of merozoites in invasion of RBCs [27]. Experimental evidence demonstrated that RAP-2 is a ligand used by merozoites to invade RBCs. This protein has four RBC binding sequences defined by high-activity binding peptides (HABPs) 26220 (^61^NHFSSADELIKYLEKTNINT^80^) and 26225 (^161^IKKNPFLRVLNKASTTTHAT^180^) in the protein’s amino terminal and central parts and HABPs 26229 (^241^RSVNNVISKNKTLGLRKRSS^260^) and 26235 (^361^FLAEDFVELFDVTMDCYSRQ^380^) in the carboxyl terminal region [25]. All HABPs bind to a 62-kDa protein located on the RBCs surface. HABPs 26225 and 26229 also bind to the 42-kDa RBC proteins.

Additionally, RAP-3 can act as an auxiliary protein in the RAP complex in maintaining the cycle during blood stage. Invasion does not become inhibited when the gene encoding RAP-3 is interrupted but does become reduced to a certain extent. RAP-3 shows two HABPs: 33860 (^61^FNHFSNVDEAIEYLKGLNIN^80^) and 33873 (^321^KNRTYALPKVKGFRFLKQLF^340^) [25].

Rhoptry-associated proteins may be used as targets for multi-antigen, multistage, subunit-based, anti-malarial vaccine development. A completely effective vaccine has not yet been developed against this disease, mainly due to the incomplete knowledge of the intimate molecular interactions between parasite proteins and their specific host cell membrane receptor(s) during invasion. The proteins identified in this study may be the starting point for more detailed studies regarding control strategies and disease prevention.

## Conclusions

In this study, analysis of immunoblotting profiles of serological reactive sera from malaria patients demonstrated a consistent pattern with respect to immunoreactivity of antigens with molecular weights in the range of 40 to 60 kDa. Between 40 and 60 kDa, it was identified four main bands in the membranes that were analysed by mass spectrometry and it was identified six immunogenic proteins: elongation factor-1 alpha (EF-1α), protein disulphide isomerase (PDI); phosphoglycerate kinase (PGK); 78 kDa glucose-regulated protein homologue (GRP-78); rhoptry-associated protein 2 (RAP 2) and rhoptry-associated protein 3 (RAP 3).

## Competing interests

The authors declare that they have no competing interests.

## Authors’ contributions

RMC, FN, KPS, RV, MSS conceived and designed the experiments; RMC, FN, RV, MSS analysed the data; RMC, KPS, MSS wrote the paper; all authors have read and approved the final manuscript.

## Supplementary Material

Additional file 1 Approval of the Ethics Committee of the Institute of Hygiene and Tropical Medicina, Lisbon – Portugal (in Portuguese). Click here for file

Additional file 2 Informed consent model used in this study (in Portuguese). Click here for file

Additional file 3 **Proteins of *Plasmodium falciparum* identified by mass spectrometry.** Click here for file
