# Supplementary material for: Immunoproteomic analysis of Plasmodium falciparum antigens using sera from patients with clinical history of imported malaria
Source: Malar J. 2013 Mar 18;12:100. doi: 10.1186/1475-2875-12-100 (PMC3605388; doi:10.1186/1475-2875-12-100)
Supplement: Additional file 1 — Approval of the Ethics Committee of the Institute of Hygiene and Tropical Medicina, Lisbon – Portugal (in Portuguese). [file 1475-2875-12-100-S1.pdf]

Parecer 4-2012-PN do CEIHMT sobre a utilização de amostras de soros disponíveis no IHMT

O Inv. Doutor Marcelo Sousa Silva apresentou um pedido de parecer sobre a utilização de amostras de soros proveniente de 500 indivíduos e disponíveis no Laboratório Central da UEI Clinica Tropical. O CEIHMT analisou o pedido e observou que:

- A proposta de utilização por fins de investigação oferece garantia de confidencialidade ainda que não descreva o processo de passagem das informações dos doentes para os investigadores com detalhes. Desta forma não oferece risco para os participantes.

- A investigação proposta pode vir a produzir um melhor entendimento da imunopatogenia da malária e da sua relação com a clínica. Para isto será eventualmente necessário obter dados da história do doente que permitiram localizar no tempo e caracterizar o(s) episódio(s) de malária.

O pedido de parecer apresentado oferece uma solução para a ausência de Consentimento Informado aquando da obtenção de sangue que é aceitável pelo CEIHMT. O CEIHMT recomenda no entanto que em caso de armazenamento para fins de investigação de biodados no futuro o Consentimento Informado do indivíduo dador seja obtido.

Com base dessas observações, o CEIHMT dá um parecer favorável e recomenda que mais adiante sejam descritos os procedimentos que garantem o anonimato.

Gilles Dussault

Presidente

Lisboa, 22 de Fevereiro 2012
