# Supplementary material for: Immunoproteomic analysis of Plasmodium falciparum antigens using sera from patients with clinical history of imported malaria
Source: Malar J. 2013 Mar 18;12:100. doi: 10.1186/1475-2875-12-100 (PMC3605388; doi:10.1186/1475-2875-12-100)
Supplement: Additional file 2 — Informed consent model used in this study (in Portuguese). [file 1475-2875-12-100-S2.pdf]

Consentimento Informado

Investigador :
Organização:
Telefone de contacto:
Título do estudo:

1.Introdução
Estamos a fazer um estudo sobre o paludismo grave nos adultos e crianças. O paludismo é uma doença causada por um parasita, através da picada de um mosquito. O nosso objectivo é avaliar adultos e crianças dos 0 aos 16 anos com diagnóstico de paludismo, para determinar alguns factores que possam contribuir para a sua gravidade e assim melhorar a compreensão da evolução da doença.
Vou-lhe dar informações sobre o estudo e pedir autorização para que você ou o seu filho/a faça parte do mesmo. Não tem de decidir hoje. Poderá pedir opiniões a outras pessoas em quem confia. Se houver palavras que não perceba, faça favor de perguntar a qualquer altura, que terei todo o prazer em responder-lhe. Se tiver questões mais tarde, poderá fazê-las a mim.

2.Descrição da pesquisa
Se concordar que você ou o seu filho/a participe no estudo, será observado/a por um médico, que fará, a si, várias perguntas para preencher um questionário com informações sobre a sua saúde ou do seu filho/filha e fará um exame físico. Quando for colhido sangue, para avaliação laboratorial da sua doença, irá colher-se uma quantidade muito pequena, que depois será enviada para Portugal para ser examinado num laboratório com métodos mais avançados de diagnóstico.

3.Riscos
Não existem riscos acrescidos para si ou o seu filho/a por participar neste estudo. Os riscos existentes, são os associados à doença e ao seu tratamento.

4.Benefícios
O sr(a)/seu filho(a) não terão qualquer benefício directo por participar neste estudo. O estudo poderá contribuir para uma melhor compreensão do paludismo e dos factores que contribuem para a sua gravidade.

5.Alternativas
O sr(a)/filho(a) não têm de participar no estudo se não quiserem. Mesmo que decidam participar, poderão, a qualquer altura, sair do mesmo. Não serão prejudicados em nada por esse facto.

6.Confidencialidade
As informações sobre você ou seu filho que constem no questionário são confidenciais e serão guardados em lugar seguro e de acesso restrito. Não partilharemos essa informação com ninguém.

7.Compensação
O sr(a)/seu filho não serão pagos por participar neste estudo. Também não terão nenhum custo suplementar por participarem no mesmo.

8.Contactos
Poderá contactar o investigador em qualquer altura durante a sua permanência no terreno e posteriormente poderão fazê-lo para o Instituto de Higiene e Medicina Tropical em Portugal, Rua da Junqueira, 100, 1349-008 Lisboa

9.Participação voluntária
A sua participação/do seu filho(a) neste estudo é inteiramente voluntária. O sr/sra é quem decide se o seu filho/a poderá participar.

10.Declaração do participante
Fui convidado para participar neste estudo. Fui informado dos procedimentos, riscos, ausência de compensações, alternativas e confidencialidade. Tive liberdade para fazer todas as questões ao investigador.

Data______________________
Nome_____________________
Assinatura ou impressão digital do Paciente ou Representante Legal

____

11. Declaração do investigador
Declaro que expliquei o estudo ao participante numa linguagem acessível, tendo este concordado em participar.

____

1ª via – Investigador
2º via – Paciente/ Representante Legal
