# Supplementary material for: Immunoproteomic analysis of Plasmodium falciparum antigens using sera from patients with clinical history of imported malaria
Source: Malar J. 2013 Mar 18;12:100. doi: 10.1186/1475-2875-12-100 (PMC3605388; doi:10.1186/1475-2875-12-100)
Supplement: Additional file 3 — Proteins of Plasmodium falciparum identified by mass spectrometry. [file 1475-2875-12-100-S3.pdf]

Table 1: Proteins of *Plasmodium falciparum* identified by mass spectrometry.

Sample	Protein Name	Accession Number	Protein MW	Protein PI	Best Peptide Sequence	Start Seq Pos	End Seq Pos	Ion Score C.I. %	Observed Mass (Da)	Match Error (ppm)
F1	*Elongation factor-1 alpha*	*gi|124513850*	*48928*	*9.1*	*VGYQADKVDFIPISGFEGDNLIEK*	*179*	*202*	*97*	*2654.35*	*4.51*
F1	*Protein disulfide isomerase*	*gi|11125364*	*55440*	*5.6*	*TPLEEFVTSESFPLFGEINTENYR*	*216*	*239*	*99*	*2819.33*	*-5.37*
F2	*Chain A, Crystal Structure Of Phosphoglycerate Kinase*	*gi|34810932*	*46321*	*7.8*	*NVQIFLPVDFK*	*285*	*295*	*99*	*1319.74*	*0.92*
F3	*78 kDa glucose-regulated protein*	*gi|121573*	*30639*	*4.7*	*SQTFSTYQDNQPAVLIQVFEGER*	*71*	*93*	*100*	*2657.40*	*4.47*
F4	*Rhoptry-associated protein 3*	*gi|21591743*	*46947*	*8.5*	*VFIFNEINFFR*	*189*	*199*	*100*	*1445.76*	*1.27*
F4	*Rhoptry-associated protein 2*	*gi|6118349*	*46732*	*8.9*	*DINPLFINDFILILNDKK*	*124*	*141*	*100*	*2145.19*	*-3.87*
F4	*Rhoptry-associated protein 2*	*gi|6118349*	*46732*	*8.9*	*AVTLYLFYYYLK*	*92*	*103*	*100*	*1556.84*	*-2.27*
